# Effect of Active Bonding Application after Selective Dentin Etching on the Immediate and Long-Term Bond Strength of Two Universal Adhesives to Dentin

**DOI:** 10.3390/polym14061129

**Published:** 2022-03-11

**Authors:** Louis Hardan, Giovana Orsini, Rim Bourgi, Carlos Enrique Cuevas-Suárez, Marco Nicastro, Florin Lazarescu, Dimitar Filtchev, Elizabeth Cornejo-Ríos, Juan Eliezer Zamarripa-Calderón, Krzysztof Sokolowski, Monika Lukomska-Szymanska

**Affiliations:** 1 Department of Restorative Dentistry, School of Dentistry, Saint-Joseph University, Beirut 1107 2180, Lebanon; louis.hardan@usj.edu.lb (L.H.); rim.bourgi@net.usj.edu.lb (R.B.); 2 Department of Clinical Sciences and Stomatology, School of Medicine, Polytechnic University of Marche, Via Tronto 10, 60126 Ancona, Italy; g.orsini@staff.univpm.it; 3 Dental Materials Laboratory, Academic Area of Dentistry, Autonomous University of Hidalgo State, Circuito Ex Hacienda La Concepción S/N, San Agustín Tlaxiaca 42160, Hidalgo, Mexico; elizabeth_cornejo@uaeh.edu.mx (E.C.-R.); eliezerz@uaeh.edu.mx (J.E.Z.-C.); 4 Private Practice, “Studio Nicastro” Dental Clinic, Corso Trieste 142, 00198 Roma, Italy; m.nicastro@mac.com; 5 Private Practice, “Trident” Dental Clinic and Postgraduate Course Centre, Street Dr. Louis Pasteur 1A, 050533 Bucharest, Romania; florin.lazarescu@clinicatrident.ro; 6 Department of Prosthetic Dental Medicine, Faculty of Dental Medicine, Medical University of Sofia, 1000 Sofia, Bulgaria; d.filchev@fdm.mu-sofia.bg; 7 Department of Restorative Dentistry, Medical University of Lodz, 251 Pomorska Street, 92-213 Lodz, Poland; krzysztof.sokolowski@umed.lodz.pl; 8 Department of General Dentistry, Medical University of Lodz, 251 Pomorska Street, 92-213 Lodz, Poland

**Keywords:** bond strength, dentin, etching, phosphoric acid, universal adhesive

## Abstract

The objective was to evaluate the influence of active bonding applications (ABA) for different time intervals after selective dentin etching (SDE) for 3 s on the microtensile bond strength (μTBS) to dentin of two universal adhesive systems (UAs): one containing 2-hydroxyethyl methacrylate (HEMA) (Scotchbond Universal, SBU), and one that was HEMA-free (Prime&Bond Universal, PBU). Dentin bovine specimens were divided into four groups: self-etch as control (SE), SDE + ABA for 15 s (SDE15), SDE + ABA for 20 s (SDE20), and SDE + ABA for 25 s (SDE25). The μTBS test was performed after a water storage of 24 h and 6 months. Scanning electron microscopy (SEM) was used in order to examine the resin–dentin interface. For the PBU, the µTBS was significantly influenced only by the aging factor (*p* < 0.026). A statistically significant decrease in the µTBS after 6 months of aging was observed only for the SDE15 group. For SBU, µTBS was significantly influenced by the protocol application and the aging time (*p* ≤ 0.041). The groups SDE15, SDE20, and SDE25 achieved statistically significant higher values (after 24 h and 6 months). No considerable variances were noticed in the homogeneity and continuity of the hybrid layer (HL) among the groups. In conclusion, SDE and ABA improved the µTBS only of a HEMA-containing universal adhesive.

## 1. Introduction

The bonding mechanism of resin composites to the enamel and dentin are slightly dissimilar [1]. Hydroxyapatite (HAp) is the essential component of enamel substrate. In addition to HAp structure, water, collagen, and a non-collagenous extracellular organic matrix are included in the dentin [2]. Since enamel and dentin are dissimilar substrates, it is imperative to recognize how they impact the performance of adhesives [3]. Resin–dentin bonding is a critical point in which the demineralization of the dentin collagen matrix used as a scaffold for resin impregnation is accomplished in order to create a stable hybrid layer (HL) [4,5]. Ideal hybridization is produced after monomer infiltration and their subsequent polymerization within the collagen network, firmly anchoring the adhesive and the overlying restoration to dentin [6]. When the HL structure ideally seals the dentin, secondary caries and post-operative sensitivity can be hindered [7]. The adhesive–dentin bond strength can be influenced by numerous factors, such as the polymerization (time, mode, exposure), the dentin region used for bonding, the composition of the adhesive system, and the application mode [8,9]. It is important to consider that the ideal hybridization results in a reliable bond strength which is essential to improve bonding effectiveness [9].

Bonding to the dentin structure is mainly micromechanical with an etch-and-rinse (ER) strategy, relying on the application for 15 s of a strong acid [10]. A phosphoric acid is the most suitable conditioner on dentin, which is believed to remove almost all the minerals and open the spatial network in the collagen matrix [11], followed by resin infiltration and in situ polymerization inside the etched surface [9]. On the other hand, the self-etch (SE) strategy does not involve a separate etching process, as these adhesives include acidic monomers in their formulation [12]. Thus, a chemical bonding occurs between functional monomers and HAp at the interface, contributing to a weaker mechanical interlocking than that of the ER strategy [13].

Universal adhesive systems (UAs) have attracted considerable research interest in recent years, as they can be used in SE, ER, and selective enamel etching (SEE) modes [14]. These adhesive systems are composed of a solution of hydrophilic and hydrophobic polymers. The hydrophilic monomers are usually transported in a water-soluble solvent (acetone, ethanol, water) to promote a good flow and penetration in the hydrophilic dentin (to influence the strength of resulting bonding), whereas the hydrophobic monomers facilitate a chemical bond with the overlying resin layer [2,3,4,5,6,7,8,9,10,11,12,13,14]. It has been established that this new type of multimode adhesive cannot fully infiltrate to the same depth of demineralized dentin produced by phosphoric acid in the ER technique [15]. Contrary to that, the HL of UAs with the SE strategy appears to be more durable, as this bonding agent comprises functional monomers capable of creating a chemical interaction with HAp and maintaining the collagen fibrils protected over time [16,17].

In an effort to increase the resin–dentin bonding, a novel method called selective dentine etching (SDE) has appeared. This approach is based on the application of phosphoric acid on the dentin surface for 3 s, which, after rinsing and drying, leaves a substrate that is moderately demineralized. This method has been recognized as a substitute to increase the bond strength of UAs to dentin over the time [18,19]. Additionally, the active application of UAs enhanced the dentin bond performance of the etching mode by enabling the infiltration of adhesives into the branches of dentinal tubules [20]. Essentially, the active application of adhesive systems by means of a scrubbing technique leads to the impregnation of a superior number of monomers inside the smear layer, thus improving the quality of the adhesive interface [21].

In this study, a new application protocol was proposed for improving dentin–resin bonding by combining two different approaches: SDE and active application. Then, the objective of this in vitro study was to evaluate the effect of active bonding application (ABA) (scrubbing the bonding for different time intervals) after SDE for 3 s on the microtensile bond strength (μTBS) of two UAs to dentin. The null hypothesis is that different time intervals of the active application of a universal adhesive after SDE has no effect on the μTBS after 24 h or 6 months of storage in distilled water.

## 2. Materials and Methods

### 2.1. Experimental Design

In this study, the μTBS of two different UAs to etched dentin was evaluated according to the following factors: (1) time intervals of active application at three levels: 15 s, 20 s, and 25 s; and (2) storage time at two levels (24 h and 6 months). Two UAs were tested: 2-hydroxyethyl methacrylate (HEMA)-containing Scotchbond Universal (SBU, 3M ESPE, St. Paul, MN, USA), and HEMA-free Prime&Bond Universal (PBU, Dentsply DeTrey GmbH, Konstanz, Germany). The adhesives applied in the SE mode were used as the control group.

### 2.2. Bonding Procedure

For the study, bovine incisor teeth were collected, washed of soft tissue, and stored for seven days in a solution of 0.5% Chloramine-T. Afterward, they were retrieved from the disinfectant solution, rinsed plentifully, and stored in distilled water at 4 °C until use. Upon the approval of the ethical committee of the faculty of dental medicine at the University of Saint-Joseph (ref.# USJ-2021-04), all these teeth were used for determining the μTBS.

For specimen preparation, the root was sectioned, and their crowns were inserted in acrylic resin, permitting the exposure of the buccal enamel surface. Subsequently, the abrasion of enamel was conducted by means of an orthodontic grinder (Essencedental, Araraquara, São Paulo SP, Brazil) until the exposure of a flat medium dentin surface. Later, the exposed dentin surface was wet-ground with #600 silicon carbide (SiC) sandpaper using a speed grinder–polisher (Buehler Ltd., Lake Bluff, Illinois IL, USA) at a motor speed of 70 rpm for 1 min under a water-cooling condition to regulate the smear layer. Then, the teeth were randomly distributed into two groups based on the adhesive system used (Table 1). Successively, 40 specimens were allocated into study groups according to the protocol application (n = 5 teeth/group): a control group where the universal adhesive was applied in SE mode, SDE + active application for 15 s (SDE15), SDE + active application for 20 s (SDE20), and SDE + active application for 25 s (SDE25).

For the SE group, the adhesive systems were applied according to the manufacturers’ instructions using the SE mode. For SDE15, SDE20, and SDE25 groups, the SDE technique was performed by applying 32% phosphoric acid gel (Scotchbond Universal Etchant, 3M ESPE) for 3 s on the dentin surface, followed by a copious rinsing with water for 15 s. Later, preparation surfaces were slightly air-dried by means of an air syringe for 5 s at least. The control of moisture was achieved using absorbent paper. Thereafter, the adhesive system was applied with active application by scrubbing the dentin surface under manual pressure for three different times: 15 s (SDE15), 20 s (SDE20), and 25 s (SDE25). The applied adhesive was gently dried for 5 s to remove the excess solvent, followed by a light curing for 20 s with a light emitting diode (LED) light-curing unit Curing Pen (Eighteeth, Changzhou, China) at 1000 mW/cm^2^.

After the bonding processes, resin composite build-ups (Filtek^TM^ Z250, 3M ESPE, St. Paul, MN, USA or Ceram X Duo, Dentsply, Konstanz, Germany) were applied in 3 increments of 2 mm each, with a polymerization for 30 s for each layer with the same light-curing device.

### 2.3. Microtensile Bond Strength Testing

The μTBS was established in accordance with ISO/TS 11405. After immersion in distilled water at 37 °C for 24 h, the specimens were divided occluso-gingivally into 1.0 mm × 1.0 mm composite dentin beams. The upper half of the beam formed the resin composite; however, the underlying dentin shaped the lower half of the beam. From each tooth, approximately ten beams were acquired, and among them, five were selected from the mid-coronal dentin and kept moist until testing. Beams in each group were randomly divided into two groups according to the μTBS testing time of 24 h and 6 months of water storage at 37 °C. The 6-month specimens were stored in distilled water with weekly replacement. Bonded resin–dentin sticks were attached to Geraldeli’s jigs using cyanoacrylate glue (Zapit, Dental Ventures of North America, Corona, CA, USA), adapted in a universal testing machine (Intron 1165, Instron, Norwood, MA, USA), and subjected to a tensile force until failure with 1 mm/min crosshead speed and 10,000 N load cell. Consequently, a cross-sectional area of each failed specimen was measured using a digital caliper with 0.01 mm of precision (Model CD-6BS Mitutoyo, Tokyo, Japan). μTBS values were expressed in MPa by dividing the force at debonding (N) by the cross-sectional surface area of the specimen (mm^2^). For each tooth, the results obtained of the five sticks tested were averaged, and the mean obtained was then used for statistical purposes. All fractured beams were mounted on aluminum stubs and examined under a stereomicroscope (40× magnification, Stereo-zoom S8, Leica, Heidelberg, Germany) in order to recognize the failure modes, which were classified as adhesive (the fracture site was within the adhesive), cohesive in composite, cohesive in dentin, or mixed (the fracture site extended into either the dentin or the resin composite).

### 2.4. Scanning Electron Microscopy Analysis

One tooth for each experimental group was restored according to the procedures described in Section 2.1. After storing at 37 °C for 24 h, the specimens were cut buccolingually and embedded in epoxy resin, allowing the dentin–resin interfaces to be visible. For scanning electron microscopy (SEM) preparation, the specimens were wet-polished with 600-, 1200-, 1500-, 2000-, and 2500-grit SiC papers followed by 3, 1, 0.25, and 0.1 μm diamond suspensions for polishing. Then, the polished surfaces were treated with 50% phosphoric acid solution for 5 s and immersed in 2.5% sodium hypochlorite (NaOCl) solution for 10 min for deproteinization. Finally, the specimens were cleaned with an ultrasonic bath for 10 min, stored at 37 °C for 2 h, and sputter-coated with gold [22]. The bonded interfaces were examined using SEM (Vega3, TESCAN, Brno, Czech Republic), at 10 kV.

### 2.5. Statistical Analysis

Data were analyzed to verify the adherence to the normality and homoscedasticity models. Then, a two-way analysis of variance (ANOVA) was conducted to verify the influence of the factors (active application time and aging) on the dependent variable. For all tests, the significance level was set at α = 0.05. All analyses carried out resulted in a power test of at least 0.8. The statistical tests were performed using Sigma Plot 12.0 software. The data concerning µTBS were statistically analyzed using a two-way ANOVA test, with the factors being: group (SE, SDE15, SDE20, and SDE25) and aging (24 h and 6 months). Each adhesive (Prime&Bond Universal (Dentsply DeTrey GmbH, Konstanz, Germany) and Scotchbond Universal (3M ESPE, St. Paul, MN, USA)) was analyzed separately.

## 3. Results

Table 2 shows the results of the µTBS of the Prime&Bond Universal adhesive (Dentsply DeTrey GmbH, Konstanz, Germany). A statistically significant decrease in the µTBS after 6 months of aging was observed only for the SDE15 group (*p* < 0.026).

Table 3 shows the results of the µTBS of the Scotchbond Universal adhesive (3M ESPE, St. Paul, MN, USA). For this adhesive, µTBS was found to be significantly influenced by the groups and aging factors (*p* ≤ 0.041), and the interaction between these factors was statistically significant, too (*p* < 0.001). The groups SDE15, SDE20, and SDE25 achieved statistically significant higher values, both at 24 h and 6 months aging than SE group. A statistically significant decrease in the µTBS after 6 months of aging was observed only for the SDE25 group.

Table 4 recapitulates the distribution of failure mode of the adhesive systems tested. For both adhesives, most of the adhesive failures were cohesive and the number of adhesive failures increased after 6 months of aging.

**Figure 1:**
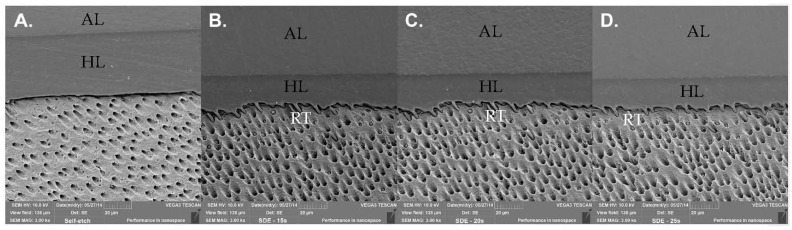
Scanning electron microscopy characterization of the bonded interfaces of the conditions tested for the application of Prime&Bond Universal adhesive (Dentsply DeTrey GmbH, Konstanz, Germany) after 24 h of aging: (**A**) self-etch method; (**B**) selective dentin etching for 15 s; (**C**) selective dentin etching for 20 s; and (**D**) selective dentin etching for 25 s. Adhesive layer (AL); resinous tags (RT); hybrid layer (HL).

**Figure 2:**
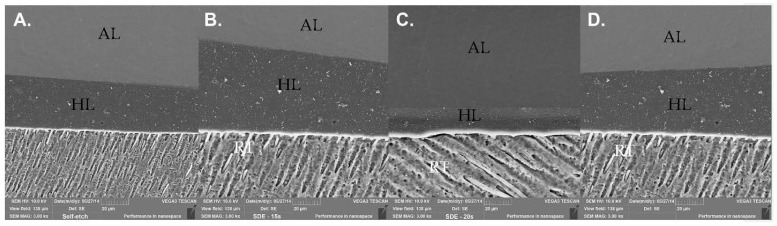
Scanning electron microscopy characterization of the bonded interfaces of the conditions tested for the application of Scotchbond Universal adhesive (3M ESPE, St. Paul, MN, USA) after 24 h of aging: (**A**) self-etch method; (**B**) selective dentin etching for 15 s; (**C**) selective dentin etching for 20 s; and (**D**) selective dentin etching for 25 s. Adhesive layer (AL); resinous tags (RT); hybrid layer (HL).

SEM images of the bonded interfaces from the different groups are presented in Figure 1 (Prime&Bond Universal adhesive, Dentsply DeTrey GmbH, Konstanz, Germany) and Figure 2 (Scotchbond Universal adhesive, 3M ESPE, St. Paul, MN, USA). No appreciable changes were noticed in the homogeneity and continuity of the HL along the interfaces among the different conditions tested. Different to the SE condition, in images where the phosphoric acid was applied, resinous tags (RT) are presents.

## 4. Discussion

In the present study, a new application protocol was proposed for improving dentin–resin bonding by combining two different approaches: SDE and active application. The adhesives selected were one containing HEMA (Scotchbond Universal, 3M ESPE, St. Paul, MN, USA) and one that was HEMA-free (Prime&Bond Universal, Dentsply DeTrey GmbH, Konstanz, Germany). The results suggest that only for the tested HEMA adhesive, the SDE, together with the active application protocol, improved the bond strength, while in the tested HEMA-free adhesive, this protocol seems to not have any influence. Hence, the null hypothesis tested in this in vitro study was partially accepted.

The durability and stability of the dentin–adhesive interface produced by these new UAs are doubtful [14]. One of the main concerns of these systems was associated with the increase in nanoleakage after aging, resulting in an inadequate bond durability [12]. HEMA is a widely used monomer in many adhesives since it helps to maintain the hydrophobic and hydrophilic monomers in a homogeneous solution, minimizing phase separation in the presence of water [22,23,24,25,26]. Although HEMA has many positive attributes, it also has disadvantages. HEMA, both in the unpolymerized and polymerized state, easily absorbs water [25]. Once polymerized, it can swell, discolor, and contribute to the hydrolysis of the adhesive interface (water blisters become entrapped in the adhesive layer) [27]. High amounts of HEMA can decrease the mechanical properties of the polymer [28]. Uncured HEMA also has the potential to lower the vapor pressure of water and can make evaporation more difficult during the air-drying step [29]. Accordingly, some dentin adhesives exclude HEMA in their composition [30]. To date, findings concerning UAs have principally focused on studying only non-etched or fully demineralized dentin as the key bonding substrates. Importantly, the capacity to use this new class of adhesives, regardless of the dentin condition, brings up unexplored opportunities concerning resin–dentin bonding to a selectively etched substrate [19]. SDE is a relatively novel method [18] used to increase the adhesion between resin and dentin by keeping HAp inside the hard-to-reach collagen spaces [19]. In summary, this study demonstrates the advantages of using 32% phosphoric acid for 3 s on the development of an ideal dentin bond of the tested HEMA adhesive. An optimum hybridization process was achieved when resin monomers infiltrated the demineralized dentin under controlled conditions [31]. This is achieved by using resin components such as HEMA, which is considered to diffuse readily into the demineralized dentin zone [32]. This is in agreement with the outcome of this study and can be described by the fact that tested HEMA adhesive benefits from the 3 s of SDE.

In addition, the application time did not have any effect on the bond strength to dentin. One should bear in mind that the application time has a positive effect on the bond strength on enamel. In general, enamel bond strength does not reach the maximum level if a short adhesive application time was used. However, a longer adhesive application time leads to a higher enamel bond strength [33]. This approach increases the interaction and diffusion of acidic resin monomers into the enamel surface, producing a more retentive pattern similar to phosphoric acid [34]. It is emphasized that unlike enamel substrate, adhesion to dentin does not depend on the etching pattern produced by acidic monomers [33]. The functional acidic monomers are able to chemically interact with HAp, and it seems that this interaction depends on the chemical structure of the specific carboxylic, phosphonic, or phosphate groups of the acidic monomers more than the time that these functional groups are able to react with the substrate [35].

Dentin etching with the active application protocol is advantageous for Scotchbond Universal (3M ESPE, St. Paul, MN, USA). This adhesive contains a polyalkenoic acid copolymer that can chemically bond to calcium in HAp [36]. More than 50% of the carboxyl groups in the polyalkenoic acid copolymer could bond to HAp. Carboxylic groups substitute the phosphate ions on the dental substrate, generating ionic bonds with calcium [37]. There is an elevated chance that the presence of a polyalkenoic acid copolymer leads to a higher bond stability between the dentin and the adhesive throughout the 6 months of storage [38]. It is expected that the presence of polyalkenoic acid copolymer favors supplementary bonding of Scotchbond Universal (3M ESPE, St. Paul, MN, USA) to dentin [23].

Furthermore, a statistically significant decrease in the µTBS after 6 months of aging was observed only for the SDE25 group. It seems that a longer application of the UAs may trigger a similar degradation process that occurs when phosphoric acid is applied [39]. This could be explained by the fact that the impregnation of the collagen fibrils exposed after demineralization may enhance the activity of matrix metalloproteinases (MMPs) and cysteine cathepsins, which encourages the degradation of the HL [30,31]. This led to the search for substances that inhibit the action of these enzymes [14]. Enhancement in the bond strength can be achieved in various manners, such as multiple layer applications [40], improved solvent evaporation [41], prolonged curing time [42], prolonged air blowing [43], using MMP inhibitors [44], crosslinking agent application [45], biomimetic remineralization [46], ethanol wet bonding [47], and SEE [48]. All in all, in this study, SDE combined with active bonding applications (ABA) of the tested HEMA adhesive was considered to be the treatment of choice when bonding to dentin substrate.

The present study noted that the SDE did not enhance the immediate and long-term bond strength of Prime&Bond Universal (Dentsply DeTrey GmbH, Konstanz, Germany) (HEMA-free adhesive). Furthermore, a significant drop in the µTBS after 6 months of aging was observed only for the SDE15 group. It is well known that the chemical composition of the adhesive system directly affects the bonding ability [27]. Additionally, the presence/absence of HEMA in an adhesive system may impact the efficacy of bonding to water-contaminated dentin under hydrostatic pulpal pressure since HEMA might prevent phase separation between the hydrophobic constituents and the diffused water from the dentinal tubules [32]. Since Prime&Bond Universal adhesive (Dentsply DeTrey GmbH, Konstanz, Germany) is a HEMA-free adhesive [30], water contamination during the bonding process may result in an initial decline in the dentin bond strength and can produce flaws among the adhesive layer and resin composite due to the phase separation between water and the hydrophobic constituents. These flaws would lead to a lessened durable bonding to dentin after 6 months of water storage [32,49]. This conclusion supports the discovery of this study since bond strength remained unchanged. In this manner, the tested HEMA-free adhesive should not be the treatment of choice when using the combined approach SDE and ABA.

Even though adequate resin–dentin bonding is commonly immediately reached, a lower bonding effectiveness develops over time [50]. This does not support the results of this study because for most cases, the bond strength of adhesives is stable after 6 months of water storage. This is clearly explained by the composition of the adhesives. It is important to mention that almost all UAs contain a functional acidic monomer known as 10-methacryloyloxydecyl dihydrogen phosphate (10-MDP), which is imperative for obtaining a stable nanolayer structure (10-MDP/Calicum salts) because it forms a chemical bond with calcium in HAp crystals [24,51]. In addition, 10-MDP monomer requires a suitable time of 20 s for the chemical reaction to take place [14]. Nevertheless, diverse application procedures are needed for those UAs with different chemical compositions, specifically when comprising an acidic functional monomer distinct from 10-MDP [43]. A crucial requirement of a dental adhesive is to form a resin–dentin bond inside the oral cavity with an appropriate durability over time in order to avoid or reduce collagen degradation and monomer hydrolysis [52]. Though manufacturers do not postulate the exact percentage of each constituent existent in the adhesives, it is potentially the case that the presence of distinctive percentages of the 10-MDP monomer can make the adhesive more or less susceptible to the degradation phenomena [23]. Indeed, 10-MDP/Calcium salts protect against hydrolysis since it is a hydrolytically stable salt, preferring the preservation of bond strength values [14,34]. Despite this, it is worth stating that for both UAs tested in this study, the increase in the number of the adhesive type of failures after 6 months of aging might suggest some type of degradation of the adhesive/dentin interface.

There are numerous different opinions regarding what to choose and which mode is more suitable to bond to dentin [40,51]. Clinicians cannot conclude that one mode is better than the other, but knowing that, nearly all UAs, including Prime&Bond Universal adhesive (Dentsply DeTrey GmbH, Konstanz, Germany) and Scotchbond Universal adhesives (3M ESPE, St. Paul, MN, USA), were based on the functional monomer 10-MDP in an attempt to achieve a stable structure (10-MDP/calcium salts) in the HL and the adhesive layer. The presence of diverse co-monomers (cross-linkers or adhesion promoters), catalysts, and solvents led to great changes in the adhesive film properties, which impacted their bond strength [23,29,30]. 

Based on the findings of this experiment, the HL along the interfaces among the different conditions tested were homogenic. Prime&Bond Universal adhesive (Dentsply DeTrey GmbH, Konstanz, Germany) and Scotchbond Universal (3M ESPE, St. Paul, MN, USA) were used in this study in an SE strategy for the control group. These UAs presented a pH of 2.5 and 2.7, respectively [29,30]. The HL is thin (0.5–1.5 μm for mild or moderate SE adhesives) compared to that which can be formed after phosphoric acid etching, which is more acidic (5 μm for ER adhesives), and which is characterized by the formation of resin tags (RT) [53]. From the SEM characterization of the bonding interface, it was noticed that the removal of the smear layer and the smear plugs by the etching step with phosphoric acid increased adhesive infiltration and facilitated the penetration of the adhesive into the dentin tubules, thus improving the tag length and morphology when compared to the SE mode [54]. Besides this, a correlation between longer RT and µTBS could not be observed in this study.

The results of this study should be considered with caution since some limitations should be acknowledged. Firstly, the clinical feasibility of applying phosphoric acid for 3 s in larger cavities might be compromised due to the time constraint. Furthermore, the smear layer was harmonized by means of 600 SiC paper, which is clinically considered as a thin layer compared to in vivo burs made by the smear layer [53]. Additionally, randomized controlled clinical trials need to be conducted to broaden the knowledge of this approach, with the aim of gaining a better understanding of the performance of UAs in the success of resin-based restorations to dentinal substrate. The UAs tested in this study are classified as mild UAs. Consequently, research should be focused towards examining other UAs with dissimilar functional monomers, in the form of a delivery, providing controlled bond strength without negotiating on material properties. Therefore, further studies should be executed to verify the current preliminary results. It must be emphasized that the key reason for the failure of dental restorations is nanoleakage produced by a hindered bond strength. Accordingly, it appears that determining a stable and durable dentin bond interface is fundamental for the long-term clinical achievement of restorative treatment [55].

## 5. Conclusions

The in vitro evidence suggests that the SDE for 3 s together with the active application protocol improved the bond strength of the tested universal adhesive containing HEMA, while in the tested HEMA-free universal adhesive, this protocol seems to not have possess any benefits.

## Figures and Tables

**Table 1:** Manufacturer and composition of the adhesives used.

Material	Classification	Composition	Manufacturer
Prime&Bond Universal (PBU)	Mild (pH = 2.5)	10-MDP, PENTA, isopropanol, water, photoinitiator, bi- and multifunctional acrylate	Dentsply DeTrey GmbH, Konstanz, Germany
Scotchbond Universal (SBU)	Mild (pH = 2.7)	10-MDP, 2-HEMA, Bis-GMA, DCDMA, MPTMS, VP-copolymer, fumed silica, ethanol, water, photoinitiators	3M ESPE, St. Paul, MN, USA

**Table 2:** Mean and standard deviation of the µTBS of the Prime&Bond Universal adhesive (Dentsply DeTrey GmbH, Konstanz, Germany).

Group	24 h	6 Months
SE	A 44.3 (7.4) ^a^	A 35.6 (5.3) ^a^
SDE15	A 48.3 (6.4 ^a^	B 38.3 (5.7) ^a^
SDE20	A 47.2 (5.6) ^a^	A 40.3 (4.7) ^a^
SDE25	A 49.6 (6.3) ^a^	A 41.4 (4.8) ^a^

Microtensile bond strength as a function of the group and aging factors. Different superscript lowercase letters indicate differences between groups after 24 h or 6 months of aging. Different uppercase letters indicate differences between 24 h and 6 months of aging for each group.

**Table 3:** Mean and standard deviation of the µTBS of the Scotchbond Universal adhesive (3M ESPE, St. Paul, MN, USA).

Group	24 h	6 Months
SE	A 45.0 (5.7) ^a^	A 38.4 (5.3) ^a^
SDE15	A 53.3 (6.4) ^b^	A 45.6 (4.2) ^b^
SDE20	A 54.2 (5.6) ^b^	A 47.9 (5.7) ^b^
SDE25	A 56.3 (6.3) ^b^	B 48.3 (4.7) ^b^

Microtensile bond strength as a function of the group and aging factors. Different superscript lowercase letters indicate differences between groups after 24 h or 6 months of aging. Different uppercase letters indicate differences between 24 h and 6 months of aging for each group.

**Table 4:** Failure pattern analysis of the bonding agents evaluated after microtensile bond strength test.

	Prime&Bond Universal				Scotchbond Universal			
	24 h		6 Months		24 h		6 Months	
	Cohesive *	Adhesive	Cohesive *	Adhesive	Cohesive *	Adhesive	Cohesive *	Adhesive
SE	61	39	42	58	52	48	36	64
SDE15	53	47	38	62	46	54	23	77
SED20	58	42	31	69	68	32	45	55
SED25	66	34	51	49	53	47	49	51

* All cohesive fractures were classified as cohesive in dentin.

## Data Availability

Derived data supporting the findings of this study are available from the corresponding author [C.E.C.-S.] on request.
